#  Multivariate Chemometrics with Regression and Classification Analyses in Heroin Profiling Based on the Chromatographic Data

**Published:** 2016

**Authors:** Slobodan B. Gadžurić, Sanja O. Podunavac Kuzmanović, Milan B. Vraneš, Marija Petrin, Tatjana Bugarski, Strahinja Z. Kovačević

**Affiliations:** a *Department of Chemistry, Biochemistry & Environmental Protection, Faculty of Science, University of Novi Sad, Trg D. Obradovića 3, 21000 Novi Sad, Serbia.*; b *Faculty of Technology, University of Novi Sad, Bulevar cara Lazara 1, 21000 Novi Sad, Serbia. *; c *National Forensic Technical Centre in Novi Sad, Pap Pavla 46, Novi Sad, Serbia. *; d *Faculty of Law, University of Novi Sad, 21000 Novi Sad, Serbia.*

**Keywords:** Illicit drug, Heroin, Forensic profiling, Multiple linear regression, Wald-Wolfowitz runs test

## Abstract

The purpose of this work is to promote and facilitate forensic profiling and chemical analysis of illicit drug samples in order to determine their origin, methods of production and transfer through the country. The article is based on the gas chromatography analysis of heroin samples seized from three different locations in Serbia. Chemometric approach with appropriate statistical tools (multiple-linear regression (MLR), hierarchical cluster analysis (HCA) and Wald-Wolfowitz run (WWR) test) were applied on chromatographic data of heroin samples in order to correlate and examine the geographic origin of seized heroin samples. The best MLR models were further validated by leave-one-out technique as well as by the calculation of basic statistical parameters for the established models. To confirm the predictive power of the models, external set of heroin samples was used. High agreement between experimental and predicted values of acetyl thebaol and diacetyl morphine peak ratio, obtained in the validation procedure, indicated the good quality of derived MLR models. WWR test showed which examined heroin samples come from the same population, and HCA was applied in order to overview the similarities among the studied heroine samples.

## Introduction

Illicit drug profiling provides law and police authorities essential physicochemical information that may assist in identification and disruption of drug trafficking in one country. Results of chemical analysis may allow investigators to determine geographical origin of the illicit drug, synthetic path and chemical precursors of synthetic drugs. The physical evidence combined with chemical analysis can be also used to establish links between different seizures of illicit drugs. This is the first attempt in Serbia to acquire chemical and profiling data on seized heroin samples and disseminate information to appropriate national and regional governmental agencies. In this paper, authors endeavored to determine the chemical fingerprints or signatures of seized heroin samples in three different locations in Serbia: border crossings: Batrovci and Horgoš, but also Novi Sad municipality, labelled as batch (1), (2) and (3). Serbia was chosen as the entering point to European Union, since the common trafficking routes from Middle East to EU are passing its territory mostly through the mentioned border crossings (1) with Croatia and (2) with Hungary. It is also known that heroin can be easily prepared at the many “homemade” laboratories, some of which have existed on the territory of Novi Sad municipality. 

Heroin is a semi-synthetic derivative of morphine. Due to differences in the way of growing opium poppies and the different synthetic routes during the synthesis of heroin, the presence and concentration of opium alkaloids vary in the final product. Also, during the acetylation of morphine, the other alkaloids of opium may react. Thus, their presence and concentrations vary in the final product. The presence of diluents (mannitol, glucose) and adulterants (caffeine, acetaminophen) provide additional information on the geographical origin of heroin and the way of production. Determining the concentration of opium alkaloids, adulterants and diluents makes chemical «profile» of heroin. Due to the presence of a large number of compounds, the chemical profiles of heroin can be very complex, which further complicates the analysis and profiling of the illicit drug (1–3).

One of the most recent ways to use large amounts of data collected during different chemical analysis of illicit drugs is the application of the chemometric tools suitable for data mining and forensic profiling. In this way, investigators can get insight of the drug production, drug trafficking and geographical origin of the sample. Chemometric approach studies are undoubtedly of a great importance in modern chemistry and biochemistry, especially because of possibility to screen a large number of chemical data (i.e. different molecules or analytes) in a short time and with a low cost (4–7). Multivariate chemometrics is a very useful and powerful tool when the main issue includes dealing with multicomponent data sets (8, 9). It allows the extraction of maximum information from complicated datasets. The conclusions in forensic science must be drawn from objective sources as much as possible. The forensic scientists must always follow rigid statistical protocols in the process of making decisions based on experimental data. Hence, our present paper explores the usefulness of multivariate chemometrics with regression and classification approaches in the discrimination of seized heroin samples. The aim of the study was to establish a simple analytical procedure followed by chemometric approach that can recover batch links among limited number of seized heroin samples.

## Material and methods

All samples used in this work were seized during various actions by the Serbian police at the border crossings (1) and (2), together with those seized on the territory of Novi Sad municipality (3). Chemical analysis was performed in the laboratories of the National Forensic Technical Center in Novi Sad. All chemicals used (chloroform, pyridine and MSTFA) were *pro analysi* quality manufactured by Merck.

**Figure 1:**
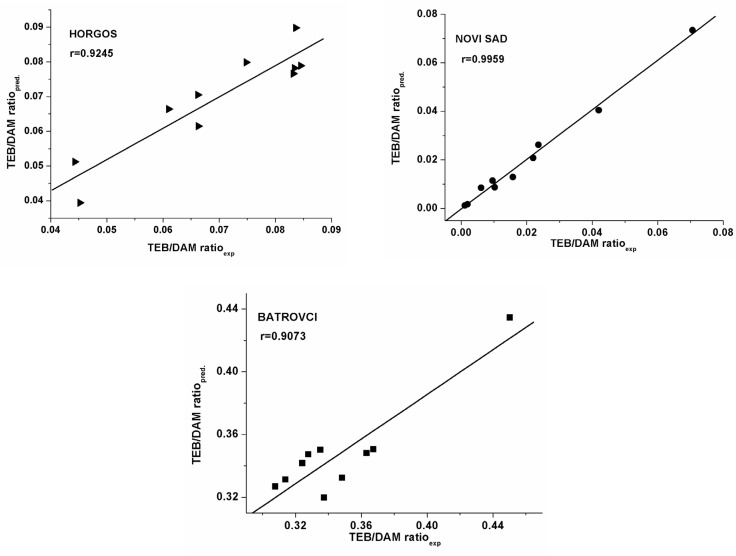
a, b, c) Plots of predicted versus experimentally observed TEB/DAM ratio

**Figure 2:**
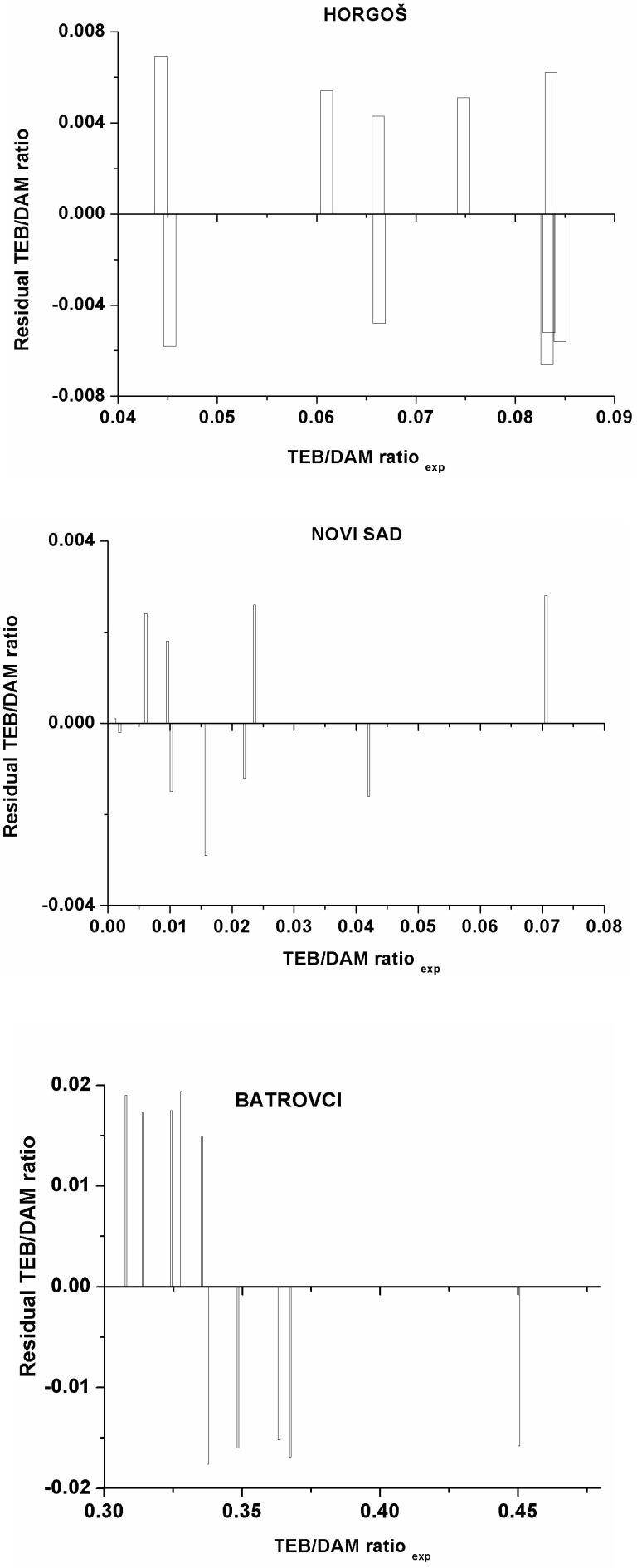
a, b, c) Plots of the residual values against the experimentally observed TEB/DAM ratio

**Figure 3:**
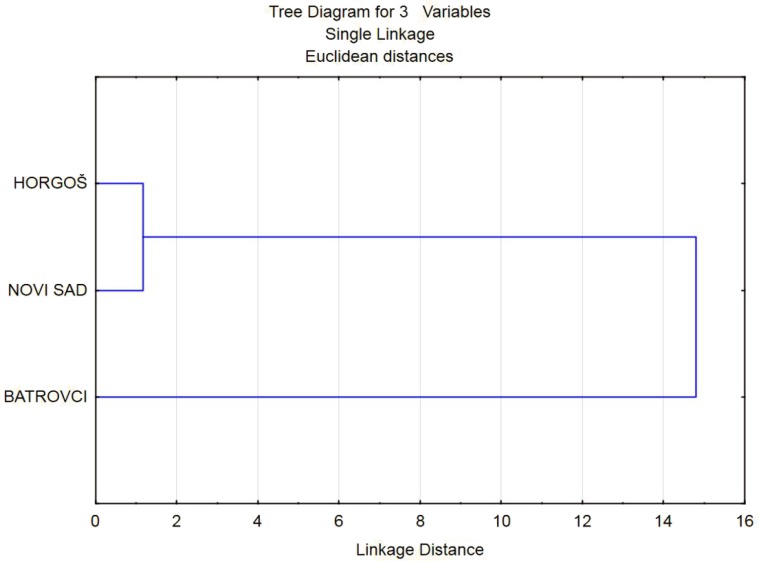
Dendrogram of HCA as a result of classification of analyzed heroin samples

**Table 1:** Peak ratio of TEB/DAM, MAM/DAM, PAP/DAM and NOS/DAM for all investigated heroin samples

	**(1)**				**(2)**				**(3)**			
	**TEB/** **DAM**	**MAM/DAM**	**PAP/** **DAM**	**NOS/** **DAM**	**TEB/** **DAM**	**MAM/DAM**	**PAP/** **DAM**	**NOS/** **DAM**	**TEB/** **DAM**	**MAM/DAM**	**PAP/** **DAM**	**NOS/DAM**
1	0.1269	0.0062	0.0424	0.1157	0.0367	0.1433	0.0479	0.0902	0.0267	0.1233	0.0379	0.0802
2	0.1562	0.0068	0.0568	0.1283	0.0411	0.1488	0.0481	0.1462	0.0311	0.1388	0.0381	0.1362
3	0.1269	0.0062	0.0424	0.1157	0.0141	0.1104	0.0246	0.1368	0.0241	0.1204	0.0346	0.1468
4	0.1562	0.0068	0.0568	0.1283	2.5076	6.7521	0.4682	0.0076	2.5276	6.7721	0.4482	0.0276
5	0.1873	0.0073	0.0492	0.1909	2.7672	7.1083	0.4746	0.0374	2.7672	7.1083	0.4746	0.0374
6	0.1524	0.0062	0.0441	0.1790	0.0135	0.0657	0.0219	0.0524	0.0135	0.0657	0.0219	0.0524
7	0.1590	0.0062	0.0452	0.1828	0.0147	0.2456	0.0327	0.0049	0.0147	0.2456	0.0327	0.0049
8	0.1566	0.0059	0.0439	0.1764	0.1103	0.1463	0.4271	0.1045	0.0103	0.0463	0.3271	0.0045
9	0.3945	0.0276	0.0474	0.1248	0.2208	0.2293	0.2335	0.3083	0.0208	0.0930	0.0335	0.1083
10	0.3652	0.0284	0.0452	0.1192	0.0248	0.0965	0.0393	0.1099	0.0208	0.0925	0.0363	0.1055
11	0.3525	0.0284	0.0455	0.1416	0.0209	0.1307	0.0303	0.1001	0.0229	0.1337	0.0363	0.1021
12	0.3577	0.0242	0.0393	0.1211	0.1143	0.2137	0.1232	0.1052	0.0143	0.1137	0.0232	0.0049
13	0.3519	0.0287	0.0465	0.1407	0.2096	0.2589	0.2229	0.2029	0.0096	0.0888	0.0229	0.0029
14	0.3449	0.0287	0.0459	0.1442	0.3101	0.4474	0.3364	0.4564	0.0101	0.1474	0.0364	0.1564
15	0.4303	0.0284	0.0485	0.1288	0.0100	0.0732	0.0279	0.1036	0.0100	0.0732	0.0279	0.1036
16	0.3261	0.0253	0.0391	0.1136	0.0243	0.1208	0.0342	0.1472	0.0243	0.1208	0.0342	0.1472
17	0.3360	0.0288	0.4460	0.1353	2.5288	6.7730	0.4499	0.0279	2.5279	6.7730	0.4489	0.0279
18	0.3378	0.0288	0.0449	0.1345	0.1140	0.1135	0.0239	0.1045	0.0140	0.1134	0.0229	0.0045
19	0.3737	0.0272	0.0454	0.1110	0.0094	0.0883	0.0225	0.0023	0.0094	0.0883	0.0225	0.0023
20	0.3432	0.0251	0.0393	0.1196	0.2150	0.2460	0.2330	0.0052	0.0150	0.2460	0.0330	0.0052
21	0.3350	0.0290	0.0459	0.1363	0.1110	0.0487	0.3276	0.1049	0.0110	0.0467	0.3276	0.0049
22	0.3149	0.0284	0.0449	0.1392	0.2470	0.1297	0.2583	0.0817	0.0270	0.1237	0.0383	0.0807
23	0.3065	0.0277	0.0445	0.1384	0.1315	0.1392	0.0386	0.1364	0.0315	0.1392	0.0386	0.1364
24	0.3261	0.0283	0.0449	0.1385	0.0292	0.0920	0.0360	0.1050	0.0202	0.0920	0.0360	0.1050
25	0.2551	0.0277	0.0453	0.1384	0.4220	0.1330	0.0360	0.1017	0.0220	0.1330	0.0360	0.1017
26	0.2719	0.0286	0.0460	0.1411	2.7975	7.1087	0.4749	0.0378	2.7675	7.1087	0.4749	0.0378
27	0.3806	0.0290	0.0478	0.1192	0.1414	0.0660	0.0224	0.0528	0.0139	0.0660	0.0224	0.0528
28	0.3496	0.0280	0.0447	0.1435	0.0299	0.0901	0.0330	0.1080	0.0200	0.0900	0.0330	0.1080
29	0.3812	0.0288	0.0463	0.1374	0.0909	0.0557	0.0215	0.0424	0.0015	0.0857	0.0319	0.0528
30	0.4047	0.0386	0.0470	0.1401	0.0296	0.0810	0.0330	0.1100	0.0772	0.2876	0.0427	0.0104
31	0.3305	0.0284	0.0488	0.1162	0.0417	0.1492	0.0376	0.1264	0.0182	0.0482	0.3071	0.0145
32	0.3621	0.0279	0.0458	0.1415	0.1003	0.1004	0.0255	0.1377	0.0072	0.1237	0.0245	0.0070
33	0.3685	0.0267	0.0466	0.1417	0.0792	0.1138	0.0342	0.1462	0.0013	0.0878	0.0329	0.0429
34	0.2921	0.0254	0.0455	0.1432	0.0713	0.1317	0.0313	0.1021	0.0368	0.1574	0.0264	0.1364
35	0.3289	0.0284	0.0465	0.1299	0.0690	0.1235	0.0339	0.1065	0.0196	0.1247	0.0483	0.0707
36	0.3063	0.0263	0.0401	0.1176	0.0710	0.0760	0.0324	0.0528	0.0288	0.1192	0.0346	0.1324
37	0.3557	0.0277	0.4360	0.1383	0.0591	0.0865	0.0393	0.1189	0.0050	0.0920	0.0440	0.1050
38	0.3666	0.0282	0.0454	0.1251	0.0768	0.1340	0.0360	0.1019	0.0109	0.0825	0.0353	0.1057

**Table 2:** Best MLR models for the prediction of heroin geographical origin

**Model**	**Y**	**Coefficient**		***N***	***R***	***S***	***F***
**(1)**	TEB/DAM	Intercept	0.1274	28	0.9270	0.0334	48.8618
		MAM/DAM	8.6544				
		PAP/DAM	-0.0353				
		NOS/DAM	-0.1806				
**(2)**	TEB/DAM	Intercept	0.0073	28	0.9919	0.0922	873.29
		MAM/DAM	0.3627				
		PAP/DAM	0.2680				
		NOS/DAM	0.1453				
**(3)**	TEB/DAM	Intercept	-0.0419	28	0.9996	0.0273	106.23
		MAM/DAM	0.3798				
		PAP/DAM	0.1073				
NOS/DAM	0.1449

**Table 3:** **The cross-validation parameters**

**Model**	**PRESS**	**SSY**	**PRESS** **/SSY**	**S**_PRESS_	***r*** ^2^_CV_	***r*** ^2^_adj_
(1)	0.0794	0.2380	0.3336	0.0532	0.6664	0.8417
(2)	0.2601	22.4969	0.0116	0.0964	0.9884	0.9898
(3)	0.0288	23.7616	0.0012	0.0321	0.9988	0.9992

**Table 4:** Predicted TEB/DAM peak ratio of test set with the residual values

**Compound**	**TEB/DAM ratio ** **predicted**			**Residuals**		
	**(1)**	**(2)**	**(3)**	**(1)**	**(2)**	**(3)**
1	0.3502	0.0799	0.0017	0.0310	0.0110	-0.0002
2	0.4345	0.0394	0.0734	-0.0298	-0.0098	0.0038
3	0.3505	0.0615	0.0114	-0.0199	-0.0198	0.0068
4	0.3417	0.0898	0.0087	0.0205	0.0105	-0.0015
5	0.3312	0.0705	0.0012	0.0373	0.0087	0.0001
6	0.3198	0.0789	0.0404	-0.0276	-0.0076	-0.0036
7	0.3481	0.0782	0.0208	-0.0192	-0.0092	-0.0012
8	0.3324	0.0766	0.0262	-0.0260	-0.0056	0.0026
9	0.3268	0.0512	0.0129	0.0290	0.0079	-0.0079
10	0.3472	0.0664	0.0085	0.0194	0.0104	0.0024

**Table 5:** The results of Wald-Wolfowitz run test for comparison of heroin samples taking into account TEB/DAM, MAM/DAM, PAP/DAM and NOS/DAM ratios together

r_cr_ = 135.94	**(1)**	**(2)**	**(3)**
**(1)**	-	H_0_ rejected (r = 65)	H_0_ rejected (r = 67)
**(2)**	H_0_ rejected (r = 65)	-	H_0_ accepted (r = 149)
**(3)**	H_0_ rejected (r = 67)	H_0_ accepted (r = 149)	-

**Table 6:** The results of Wald-Wolfowitz run test for comparison of heroin samples taking into account TEB/DAM, MAM/DAM, PAP/DAM and NOS/DAM ratios separately

	**(1)**	**(2)**	**(3)**
**TEB/DAM**	r_cr_ = 30.51		
**(1)**	-	H_0_ rejected (r = 11)	H_0_ rejected (r = 3)
**(2)**	H_0_ rejected (r = 11)	-	H_0_ rejected (r = 28)
**(3)**	H_0_ rejected (r = 3)	H_0_ rejected (r = 28)	-
**MAM/DAM**	r_cr_ = 30.51		
**(1)**	-	H_0_ rejected (r = 2)	H_0_ rejected (r = 2)
**(2)**	H_0_ rejected (r = 2)	-	H_0_ accepted (r = 48)
**(3)**	H_0_ accepted (r = 48)	H_0_ rejected (r = 2)	-
**PAP/DAM**	r_cr_ = 30.51		
**(1)**	-	H_0_ rejected (r = 9)	H_0_ rejected (r = 11)
**(2)**	H_0_ rejected (r = 9)	-	H_0_ accepted (r = 40)
**(3)**	H_0_ rejected (r = 11)	H_0_ accepted (r = 40)	-
**NOS/DAM**	r_cr_ = 30.51		
**(1)**	-	H_0_ rejected (r = 13)	H_0_ rejected (r = 10)
**(2)**	H_0_ rejected (r = 13)	-	H_0_ accepted (r = 41)
**(3)**	H_0_ rejected (r = 10)	H_0_ accepted(r = 41)	-

*Analytical procedure.*

Heroin samples were homogenized first in a mortar. Mass of 0.15–0.25 g of the sample was quantitatively transferred to vials, together with 200 μL of chloroform + pyridine (1:1) solution in order to dissolve the samples and 200 μL of silylating reagent (MSTFA). Prepared samples are heated for 1 h at 60 °C and then injected in the gas chromatograph with flame ionization detector GC-FID Agilent 6890N. Injected volume was 2 μL and split mode 50:1. Chromatographic separation was achieved on a capillary column DB-1 (length 30 m, internal diameter 0.25 mm, film thickness 0.25 μM). Carrier gas was nitrogen at a pressure of 66.6 kPa. The samples were heated for one minute at 150 °C, then up to 250 °C with heating rate of 10 ºC min^–1^. This temperature was maintained for 10 min.

*Statistical Methods.*

The complete regression and classification analyses (MLR, HCA, WWR test) were carried out by PASS 2005, GESS 2006, NCSS Statistical Software, MS Excel and Statistica v. 10 (10–12).

The general purpose of MLR analysis is to quantitate the relationship between several independent or predictor variables and a dependent variable. MLR model is built with descriptive variables using the least squares methods to minimize the residuals (13). General MLR model is:

*y* = *a* + *b*_1_∙x_1_ + *b*_2_∙x_2_ +∙∙∙+ *b*_n_∙x_n_                    (1)

where *y* is the quantitative property to predict (dependent variable), x_n_ an independent (descriptive) variable, *a *the intercept, and *b*_n_ the regression coefficient for x_n_.

HCA is a method for dividing a group of objects into classes so that similar objects are in the same class (cluster). The groups of entities are not known prior to the mathematical analysis and no assumptions are made about the distribution of the variables. Cluster analysis searches for objects which are close together in the variable space. The data in each cluster share some common trait, often proximity according to some defined distance measure (14).

Wald-Wolfowitz run test (WWR) can be applied to examine if two random samples come from populations with the same distribution. WWR test can detect differences in averages or spread or any other important aspect between the two populations (15). This test is efficient when each sample size is greater than or equal to 10 (15). This method includes testing the null hypothesis - H_0_: two samples come from populations having the same distribution. At the start it is necessary to define the critical value for “run” number (r_cr_). We can calculate this value based on the following equations (15):

r_cr_ = μ – 1.96 σ (at 5% level of significance)                     (2)

where: 

μ = 1 + ((2 n_1_ n_2_) / (n_1_ + n_2_))                     (3)

σ = **(**(2 n_1_ n_2_ (2 n_1_ n_2_ – n_1_ – n_2_)) / ((n_1_ + n_2_)^2^ (n_1_ + n_2_ – 1))**)**^½^                     (4) 

n_1_ – size of sample 1

n_2_ – size of sample 2

So-called “run” number (r) can be obtained from the list of n_1_ + n_2_ observations from two samples in order of magnitude. It represents the number of sections of consecutive values which belong to the same sample and it can be counted from the list of n_1_ + n_2_ observations. Observations from sample 1 should be denoted as *X*s and other as *Y*s, and then the number of runs can be counted. Afterwards, the r and r_cr_ numbers can be compared. The H_0_ hypothesis has to be rejected if r ≤ r_cr_ (15).

## Results and discussion

In the first step of the present study, gas chromatography (GC) analyses were applied on thirty eight different samples of heroin from three locations in Serbia. The data of gas chromatography (GC) analyses are summarized in Table 1. as the peak area ratio of four secondary components, namely: acetyl thebaol (TEB), 6-monoacetyl morphine (MAM), papaverine (PAP), noscapine (NOS), and the main psychoactive component of diacetyl morphine (DAM). All these components were identified according to their retention times. In the second step, we focused our efforts on developing the MLR models that can determine the geographical origin of heroin samples. A set of twenty eight collected data (samples 1-28) was used for MLR modeling. TEB/DAM was used as a dependent variable in the regression analysis, and MAM/DAM, PAP/DAM and NOS/DAM were used as independent variables.

MLR procedure was used to model the relationships between the data of GC analyses. The stepwise regression (SWR) method was used to derive the most significant models as a calibration models for prediction of TEB/DAM peak ratio of seized heroin samples. The specifications for the best selected MLR models are shown in Table 2.

The statistical quality of the generated models was checked by statistical parameters: correlation coefficient (*r*), standard error of estimation or standard deviation (*s*), and *F*-test (Fisher›s value) for statistical significance (16–18). Correlation coefficient *r *(or coefficient of multiple determination) is a relative measure of the fit by the regression equation. Correspondingly, it represents the part of variation in the observed data that is explained by the regression. Standard deviation is measured by the error mean square, which expresses the variation of the residuals or the variation from the regression line. Thus, standard deviation is an absolute measure of the quality of the fit and should have a low value for the regression to be significant. The *F*-test reflects the ratio of the variance explained by the model and the variance due to the error in regression. High value of the *F*-test indicates that the model is statistically significant. 

It is well known that there are three important components in any chemometric-regression analysis: the development of the models, validation of the models and the utilization of developed models. Validation is a crucial aspect of any regression analysis (19). For testing the validity of the predictive power of selected models leave one out (LOO) technique was used. The developed models were validated by the calculation of the following statistical parameters: PRESS, SSY, S_PRESS_*, r*^2^_CV_, and *r*^2^_adj_ (Table 3.). These parameters were calculated from the following equations:

$\mathrm{PRESS}=\sum {\left(Y_{\mathrm{obs}}-Y_{\mathrm{calc}}\right)}^{2}$

(5)

$\mathrm{SSY}=\sum {\left(Y_{\mathrm{obs}}-Y_{\mathrm{mean}}\right)}^{2}$

(6)

$S_{\mathrm{PRESS}}=\sqrt[{2}]{\frac{\mathrm{PRESS}}{n}}$

 (7)

$r_{\mathrm{cv}}^{2}=1-\frac{\mathrm{PRESS}}{\mathrm{SSY}}$

(8)

$r_{\mathrm{adj}}^{2}=1-(r^{2})\left(\frac{n-1}{n-p-1}\right)$

(9)

where, *Y*_obs_, *Y*_calc_ and *Y*_mean_ are observed, calculated and mean values; *n* is number of the samples and *p* is number of independent parameters.

PRESS is an acronym for prediction sum of squares. It is used to validate a regression model regarding to its predictability. To calculate PRESS, each observation is individually omitted. The remaining *n*-1 observations are used to calculate a regression and estimate the value of the omitted observation. This is done *n* times, once for each observation. The difference between the actual *Y* value, *Y*_obs_, and the predicted *Y*_calc_, is so-called the prediction error. The sum of the squared prediction errors is the PRESS value. The smaller PRESS is, the better predictability of the model is achieved. SSY are the sums of squares associated with the corresponding sources of variation. These values are in terms of the dependent variable, *Y*. 

The above PRESS value can be used to compute an *r*^2^_CV_ statistic, called *r*^2 ^cross validated parameter, which reflects the prediction ability of the model. This is a good way to validate the prediction of a regression model without selecting another sample or splitting the data. It is very possible to have a high *r*^2^ and a very low *r*^2^_CV_. When this occurs, it implies that the fitted model is data dependent. This parameter ranges from below zero to above one. When outside the range of 0-1, it is truncated to stay within this range. Adjusted *r*-squared (*r*^2^_adj_) is an adjusted version of *r*^2^. The adjustment seeks to remove the distortion due to a small sample size.

In many cases *r*^2^_CV_ and *r*^2^_adj_ are taken as a proof of the high predictive ability of MLR models. A high value of these statistical characteristics (>0.5) is considered as a proof of the high predictive ability of the model. However, some recent reports have proved the opposite (20). Although, the low value of *r*^2^_CV_ for the training set can indeed serve as an indicator of a low predictive ability of a model, the opposite is not necessarily true. Thus, the high value of LOO *r*^2^_CV_ is the necessary condition for a model to have a high predictive power, but it is not a sufficient one. 

Although models showed good internal consistency, they may not be applicable for the analogs which were never used in the generation of the correlation. It is proven that the only way to estimate the true predictive power of a model is to test it on a sufficiently large collection of the samples from an external test set. The test set must include no less than five samples, whose properties and structures must cover the range of properties and structures of the samples from the training set. This application is necessary for obtaining trustful statistics for comparison between the observed and predicted values for these compounds. Therefore, the external extrapolation power of the model was further authenticated by a test set of ten heroin samples.

The values of TEB/DAM peak ratio of an external set of heroin samples (samples 29-38) were calculated by the models. These data are compared with experimentally obtained values of TEB/DAM ratio (Table 4. Figure 1.). From the data presented in Table 4. it is shown that high agreement between experimental and predicted TEB/DAM ratio was obtained (the residual values are small, indicating the good predictability of the established models). According to the reference (16) without the validation of the MLR models by using the external test set, we could not come to a right conclusion about high predictive ability of derived models.

To investigate the existence of a systemic error in developing the MLR models, the residuals of predicted TEB/DAM peak ratio values were plotted against the experimental values in Figure 2. The propagation of the residuals on both sides of zero indicates that no systematic error exists in the development of regression models as suggested by Jalali-Heravi *et al*. (21). It indicates that these models can be successfully applied to predict the geographic origin of seized heroin samples using the GC results. Therefore, the randomness of the residuals and their low values indicate that the obtained mathematical models can predict the dependent variable with acceptable error. According to the Variance Inflation Factor (VIF), which was lower than 10 for all the obtained models, it can be concluded that there is no multi collinearity present in the established models.

HCA was performed on the TEB/DAM, MAM/DAM, PAP/DAM and NOS/DAM peak ratios of the analysed heroin samples in order to reveal the similarities among them. Clustering was based on the Euclidean distance and single linkage algorithm. The obtained dendrogram is presented in Figure 3. As it can be seen from the presented dendrogram, on the basis of TEB/DAM, MAM/DAM, PAP/DAM and NOS/DAM peak ratios, the most similar heroin samples come from border crossing (2) and Novi Sad municipality (3).These entities are placed into the separate cluster, while the samples that belong to border crossing (1) are significantly different than the others.

WWR test was applied firstly on the TEB/DAM, MAM/DAM, PAP/DAM and NOS/DAM peak ratios together. The established null hypothesis “two samples come from populations having the same distribution” was confirmed for the samples that are seized at border crossing (2) and Novi Sad municipality (Table 5). This result confirms the finding obtained with HCA method. According to WWR test, the samples from Novi Sad municipality and border crossing (1) do not belong to the same population, as well as the samples from border crossings (1) and (2).

Testing the H_0_ hypothesis for the examined samples on the basis of TEB/DAM, MAM/DAM, PAP/DAM and NOS/DAM peak ratios separately, resulted in the same way as in previous WWR analysis, except in the case which included TEB/DAM peak ratio (Table 6). In this case, all the three types of samples do not belong to the same population. It can indicate that exactly TEB/DAM peak ratio can be used as discriminating factor for the analysed samples. As it is shown in the MLR analysis, this ratio actually is dependent variable which is predicted based on the other determined peak ratios.

## Conclusion

Collected data were modeled by MLR, HCA and WWR methods. Mathematical dependences that can determine the geographical origin of heroin samples were obtained. The validity of the models has been evaluated by the determination of suitable statistical parameters. Predictive ability of defined mathematical model was tested by comparing and correlating the experimental and calculated values of TEB/DAM peak ratio. The low residual activity and high cross-validated *r*^2^ values (*r*^2^_CV_) indicated the predictive ability of the developed MLR models. Since the correlation was extremely good, our mathematical models can be used to predict geographic origin of seized heroin samples in Serbia, using the GC results. HCA analysis showed that the samples from Novi Sad municipality and border crossing (2) are very similar, while WWR testing explained that mentioned samples belong to the same population according to MAM/DAM, PAP/DAM and NOS/DAM peak ratios. TEB/DAM peak ratio (the dependent variable in MLR model) was discriminating factor in WWR testing and it showed that the analysed samples do not belong to the same population.
